# Exploring movement entrainment in an ecologically valid concert setting

**DOI:** 10.1038/s41598-025-13376-7

**Published:** 2025-08-29

**Authors:** Maren Hochgesand, Hauke Egermann

**Affiliations:** Cologne Systematic Musicology Lab, Institute of Musicology, University of Cologne, Cologne, Germany

**Keywords:** Movement entrainment, Live concert, Popular music, Dance, Psychology, Human behaviour

## Abstract

Moving to the beat is one of the most natural human reactions while listening to music and can be defined as movement entrainment. Listeners’ reactions to music have been discussed as the result of the music, the context, and the listener. However, as music is a social phenomenon typically performed in live settings, movement entrainment and its contributing factors have never been investigated in ecologically valid concerts. In a preliminary study (*n* = 42) during a rock/pop concert, we confirmed the validity of our method of measuring acceleration data of the audience with portable devices on the arm and torso. In our main study (*n* = 69), we measured audience data at two big band concerts with different conditions: One was seated, the other unseated. With several consecutive Hierarchical Linear Models, we identified factors predicting movement entrainment: location of measurement (arm vs. torso), metrical level (BPM vs. half- and double-time level), musical factors (the different pieces), concert condition (seated vs. unseated), and the personal characteristic of urge to dance. Our study shows that there are multiple factors contributing to movement entrainment and can be seen as a first step towards studying this phenomenon in ecologically valid concert settings.

**Supplementary Information:**

The online version contains supplementary material available at 10.1038/s41598-025-13376-7.

## Introduction

Music makes people move, whether we are in a club, doing a waltz, or simply listening to a song on the radio, music can make us dance spontaneously. Moving our body to the beat is one of the most natural human reactions while listening to music. Typical reactions are head nodding, feet tapping, rocking along, or even dancing^1^. Some people just feel the need to move in synchrony with the music^2,3^, and some pieces just make people want to move. The song *In the Stone* by Earth Wind and Fire, which will play a role in this paper, was once described as an “irresistible dancing cut” that “moves to a swinging mixture of catchy percussives and intricately woven harmonies” in a contemporary music magazine^4^. This designation refers not only to the danceable quality of the piece, but also to the impact that music exerts on dancing behavior. In some cultures, music and dance are no separable cultural categories at all^5^ and this close connection between music and dance has led to the hypothesis that the two have evolved together over time^6,7^.

The synchronization of our body to music can be defined as movement entrainment. Entrainment has been described as the process in which two (or more) independent rhythmical, biological, or mechanical systems attune each other resulting in coordinated rhythmic behavior^2,8^. For humans, this can be synchronization with another person, for example, or with an external stimulus such as music. Music contains numerous recurring structures (e.g., a beat structure) that allow listeners to perceive a periodic stable pattern and to synchronize their movements to it^9^. The ability to synchronize movements with a musical beat has mostly been investigated with finger tapping studies (for a review, see^10,11^).

Research suggested that there is a preferred tempo of 120 beats per minute (BPM), as at this specific tempo the perception of tempo is considered to be optimal and appears most natural^12^. In this context, it is possible to draw links to the natural human walking tempo, as the spontaneous duration of steps was found to be around 500–550 milliseconds, which corresponds to a tempo between 110 and 120 BPM^13–16^. According to these considerations, it could be assumed that music with tempi between 110 and 120 BPM stimulates more movement entrainment – i.e. synchronous movement to the beat of the music – than music with slower or faster tempi. However, there is also evidence that the tempo of the music has no influence on movement features of participants^17^.

While listening to music, we often perceive not just one isochronous pulse, but several beat levels. The organization of these beat levels is typically hierarchical, wherein one level can be described as an integer multiple of the other level^18^. The interaction between these different beat perceptions leads to the perception of a periodic alternation of strong and weak beats, which corresponds to the generally accepted definition of musical meter^19^. The metrical structure usually derives from the accent structure of the music, which in turn is derived from various characteristics such as loudness, duration, and the pitch of single tones or changing harmonies^20^. Most Western music has either a duple (accent on every second beat) or a triple meter (accent on every third beat)^20^. Research focusing on synchronization of movements to different metrical levels found that participants embody different metrical levels of musical stimuli with different body parts^17,20,21^. This bodily synchronization to music can already be found in infants^22^. The observation that even young children react to music and metrical stimuli with more rhythmic movements than to speech indicates that humans have a predisposition for rhythmic movements to music and regular metrical sounds.

Music is a widespread social phenomenon, typically performed and experienced in live settings^23,24^. Therefore, we claim that one of the most suitable settings to study movement entrainment would be a live concert. However, most of the previous research studied body movement and dancing rather in laboratory studies^17,21,25–28^ or in settings with atypical small groups^29–32^. A study investigating head movements during a rock concert seated their participants^33^ which might have resulted in limited movement and did not allow for dancing. Some research has already been conducted in concerts of Western classical music to investigate audiences’ physiological synchrony^34,35^. Tschacher et al.^35^ further report synchronized movement of the audience members seated in rows, measured via pixel changes in video recordings taken from birds-eye cameras. They found that movement synchrony – conceptualized as interpersonal synchrony^36^, not synchronous movement to the music – was not associated with listeners’ personality traits, affectivity, and aesthetic experiences. In consideration of these studies, the aim of this article is to investigate the phenomenon of movement entrainment for the first time in an ecologically valid concert setting that allows people to dance and move freely. Therefore, we chose the genres of rock, pop, and jazz music, as concerts in this field of music welcome and desire audience movements.

Listeners reactions to music have been discussed as the result of the music, the context, and the listener by various researchers: Scherer and Zentner^37^ propose that the emotional responses to music are influenced by four primary factors: structural features, performance features, listener features, and contextual features. Proposing a similar reciprocal-feedback model of musical response, Hargreaves^38^ describes how the music, the situation and context, and the listener interact and finally lead to a response. The importance of the listening situation on musical preference was emphasized by North and Hargreaves^39^.

The aesthetic experience of music in a concert is presented as the encounter of an individual person with a piece of music (sound) in a specific situation or concert frame^40^. The concert frame encompasses environmental, aesthetic, social and situational properties of a concert. This could include the venue, staging, lighting and sound, for example.

It is therefore reasonable to conclude that several factors, dependent on the music itself, the context or concert frame, and the listener contribute to the phenomenon of movement entrainment. Research has examined effects of musical factors on movement to the music and movement synchrony. It was found that certain musical passages of electronic dance music (EDM) increase beat synchronisation and group synchronization of dancers in an club setting^29,30^ and it was also found that EDM evokes more movement than genres like Latin, Funk, and Jazz^28^. Musical features of pulse clarity, high spectral flux, percussiveness, bass frequencies, and moderate degrees of syncopation are also known to evoke more movement at least in certain parts of the body^17,27,41,42^.

The impact of specific components of the concert frame on concert experience has lately been studied in the context of Western classical music^43^. This research highlights the importance of the concert frame and how situational factors influence the concert experience.

In addition to the musical characteristics and the context, there are also indications that personal characteristics of the listeners have an effect on movement entrainment. It is likely that people with a higher degree of dance sophistication^44^ will move and dance more. It was further found that people in a positive mood move more, faster, and with more complex movements than other people^25^, and another study suggests a positive association of trait empathy and rhythmic entrainment while moving to music^45^. Further research observed more vigorous head movements of fans of the playing band than of neutral listeners during a concert and that fans were also more entrained to the music^33^, which suggests that liking a band or the music they play might increase movement entrainment. The positive evaluation of experiencing the musicians could also contribute to such a positive evaluation. Another study in a club also found a positive association between movement and familiarity with the music^46^. We are interested in how these factors, which we will refer to as *personal factors*, contribute to movement entrainment in an ecologically valid concert setting.

It is likely that a combination of musical, situational, and personal factors influence intensity of movement entrainment in real-world settings like concerts.

**Fig. 1:**
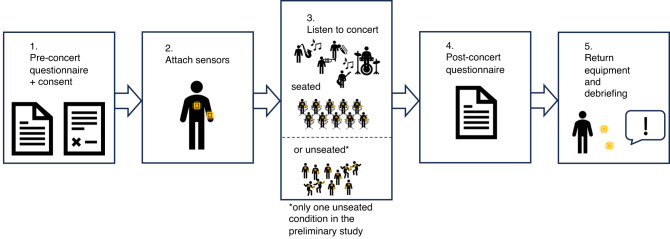
Overview of the experimental procedures.

As our research is the first to measure movement entrainment and its predictors in an ecologically valid concert setting, our initial aim was to develop a suitable method for this measurement. We used small, portable devices to measure acceleration data, which we attached to the arms and torsos of our participants in the audience. With these devices, participants were able to move freely. We defined movement entrainment as the extent to which people moved to the beat of the music they heard in the concert. To approach and test this method, we first conducted a preliminary study at a rock/pop concert. In our main study, we added an experimental condition to introduce more variance into movement entrainment. We measured audiences’ movement entrainment at two similar big band concerts, with the only difference that one concert was seated, and the other concert was not seated and people had to stand. Before and after the concert, we assessed concert experience and *personal factors* via questionnaires. Figure 1 provides an overview of the design of both studies. The following research questions drove our research:

(RQ_1_). Can movement entrainment be measured in an ecologically valid concert setting?

After having tested the measuring method in the preliminary study, we applied it to our main study and asked:

(RQ_2_). Which factors do influence movement entrainment?

We hypothesized that we would observe varying degrees of movement entrainment depending on *factors of measurement* – i.e., effects of the location of measurement and the metrical level. We also expected that *musical factors* would have an impact on movement entrainment. We have limited our analysis to the effects of tempo as a characteristic musical feature^13–16^ and have included further musical features by considering the individual pieces in the analysis. Additionally, we hypothesized that we would observe more movement entrainment in the standing condition compared to the sitting condition (*situational factor*). Finally, we expected effects of different *personal factors*: dance sophistication^44^, positive affective state^25^, trait empathy^45^, liking the concert or the played music^33^ and the evaluation of experiencing the musicians live, and being familiar with the music^46^.

## Results

### Preliminary study

We measured movement entrainment of 42 participants during a concert with several bands, with around 100 people in the audience in total. Participants filled out pre- and post-concert questionnaires to report sociodemographic data.

To validate our measurement of movement entrainment, we compared the mean values of movement entrainment averaged over all pieces for the target BPM values to corresponding values of movement entrainment calculated for control BPM values. We set the control BPM values at + 10, + 20 and + 30 BPM above the target BPM values, and − 10, − 20 and − 30 BPM below it. We analyzed movement entrainment for each of these control BPM values and took the mean.

**Fig. 2:**
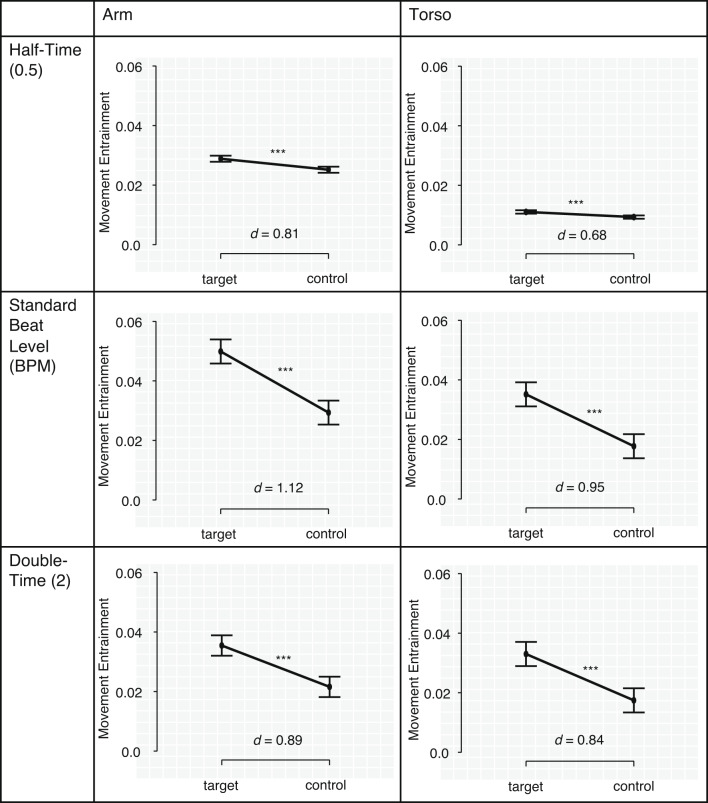
Mean values of movement entrainment calculated for the target BPM values of all pieces compared to values for movement entrainment calculated for control BPM values. Results for the two locations of measurement (arm and torso) and the three metrical levels (half-time level, standard beat level and double-time level) are shown in the six plots. For the control values, movement entrainment was analyzed for + and − 10, 20 and 30 BPM around the target value, then averaged. Error bars mark 95% confidence intervals. ***p < 0.001. Effect sizes represent differences between movement entrainment calculated for target and control BPM values.

In this comparison, we differentiated between the two locations of measurement (arm and torso) and the three metrical levels (half-time level, standard beat level, double-time level), therefore we had 12 entrainment values for every participant (six each for the target BPM values and for the control BPM values). After Bonferroni corrections for six paired *t*-tests (α = 0.0083) we found that participants’ mean value of movement entrainment calculated for the target BPM values was always significantly higher compared to movement entrainment calculated for the control BPM values, *t*(41) > 4.48, *p* < 0.001 (Fig. 2).

We further found that the mean value of movement entrainment during the concert correlated significantly and positively with participants’ self-reported desire to move and dance during the concert from the post-concert questionnaire (*r*(42) = 0.397, *p* = 0.009). This also confirms the validity of our measurement of movement entrainment and shows that the actual behavior and the self-reported desire to dance during the concert are congruent.

### Main study

We conducted the main study during two big band concerts with a total of 69 complete data sets of participants who attended one of the two concerts. The concerts had the same program, with only one variation: Concert 1 was seated and Concert 2 was unseated. We assessed personal characteristics and concert experience with pre- and post-concert questionnaires.

To investigate the effects of *factors of measurement*, *musical factors*, the *situational factor*, and *personal factors* on the dependent variable *movement entrainment*, we fitted several Hierarchical Linear Models in a forward fitting approach. We used the lmer function from the lme4 package^47^ in R^48^. All continuous variables were z-standardized.

#### Factors of measurement

First, we included the *factors of measurement*, i.e. the location of measurement *arm* (vs. torso), and the metrical levels *half-time level* (vs. standard beat level) and *double-time level* (vs. standard beat level) (M1) which improved the model fit compared to the null model (M0). This was assessed using likelihood ratio tests (Table 1). Movement entrainment measured at the arm was significantly higher compared to the measurement at the torso, *t*(3287) = 12.04, *p* < 0.001. Additionally, the highest degree of movement entrainment was measured at the standard beat level. There was less movement entrainment at the double-time level, *t*(3287) = −7.34, *p* < 0.001, and even less movement entrainment at the half-time level, *t*(3287) = −9.65, *p* < 0.001 (Supplementary Table S1).

**Table 1:** Hierarchical linear model for the dependent variable movement entrainment investigating the effects of factors of measurement, musical factors, a situational factor, and personal factors as independent variables.

	Model	AIC	BIC	Marginal *R*^2^	Conditional *R*^2^	Model fit improvement	
						χ^2^ (df)	*p*
M0	Movement Entrainment Null Modell	8735	8754		0.259		
M1 (vs. M0)	Movement Entrainment ~ Arm (vs. Torso) + Half-Time Level (vs. Standard Beat Level) + Double-Time Level (vs. Standard Beat Level)^1^	8503	8540	0.051	0.311	238.00 (3)	< 0.001
M2 (vs. M1)	Movement Entrainment ~ Arm (vs. Torso) + Half-Time Level (vs. Standard Beat Level) + Double-Time Level (vs. Standard Beat Level) + Piece^1^	8148	8277	0.122	0.384	369.44 (7)	< 0.001
M3 (vs. M1)	Movement Entrainment ~ Arm (vs. Torso) + Half-Time Level (vs. Standard Beat Level) + Double-Time Level (vs. Standard Beat Level) + Tempo	8473	8515	0.057	0.318	32.70 (1)	< 0.001
M4 (vs. M1)	Movement Entrainment ~ Arm (vs. Torso) + Half-Time Level (vs. Standard Beat Level) + Double-Time Level (vs. Standard Beat Level) + Slower than Sweet Spot + Faster than Sweet Spot	8428	8477	0.067	0.334	78.88 (2)	< 0.001
M4a (vs. M1)	Movement Entrainment ~ Arm (vs. Torso) + Half-Time Level (vs. Standard Beat Level) × Faster than Sweet Spot (vs. Sweet Spot) + Double-Time Level (vs. Standard Beat Level) × Slower than Sweet Spot (vs. Sweet Spot)	8427	8488	0.068	0.338	48.28 (4)	< 0.001
M5 (vs. M2)	Movement Entrainment ~ Arm (vs. Torso) + Half-Time Level (vs. Standard Beat Level) + Double-Time Level (vs. Standard Beat Level) + Piece^1^ + Standing (vs. Sitting)	8131	8217	0.188	0.384	19.00 (1)	< 0.001
M6 (vs. M5)	Movement Entrainment ~ Arm (vs. Torso) + Half-Time Level (vs. Standard Beat Level) + Double-Time Level (vs. Standard Beat Level) + Piece^1^ + Standing (vs. Sitting) + Dance Sophistication (Urge to Dance + Body Awareness + Dance Training + Observational Dance Experience + Social Dancing)	8132	8248	0.213	0.384	8.93 (5)	0.112
**M7 (vs. M5)**	**Movement Entrainment ~ Arm (vs. Torso) + Half-Time Level (vs. Standard Beat Level) + Double-Time Level** **(vs. Standard Beat Level) + Piece**^1^ **+ Standing (vs. Sitting) + Urge to Dance**	**8125**	**8216**	**0.211**	**0.384**	**8.16 (1)**	**0.004**
M8 (vs. M7)	Movement Entrainment ~ Arm (vs. Torso) + Half-Time Level (vs. Standard Beat Level) + Double-Time Level (vs. Standard Beat Level) + Piece^1^ + Standing (vs. Sitting) + Urge to Dance + Positive Activation	8125	8223	0.214	0.384	1.45 (1)	0.229
M9 (vs. M7)	Movement Entrainment ~ Arm (vs. Torso) + Half-Time Level (vs. Standard Beat Level) + Double-Time Level (vs. Standard Beat Level) + Piece^1^ + Standing (vs. Sitting) + Urge to Dance + Trait Empathy	8126	8224	0.213	0.384	0.94 (1)	0.033
M10 (vs. M7)	Movement Entrainment ~ Arm (vs. Torso) + Half-Time Level (vs. Standard Beat Level) + Double-Time Level (vs. Standard Beat Level) + Piece^1^ + Standing (vs. Sitting) + Urge to Dance + Liking the Concert	8127	8225	0.211	0.384	0.09 (1)	0.077
M11 (vs. M7)	Movement Entrainment ~ Arm (vs. Torso) + Half-Time Level (vs. Standard Beat Level) + Double-Time Level (vs. Standard Beat Level) + Piece^1^ + Standing (vs. Sitting) + Urge to Dance + Liking Jazz Music	8126	8224	0.212	0.384	0.48 (1)	0.488
M12 (vs. M7)	Movement Entrainment ~ Arm (vs. Torso) + Half-Time Level (vs. Standard Beat Level) + Double-Time Level (vs. Standard Beat Level) + Piece^1^ + Standing (vs. Sitting) + Urge to Dance + Being Familiar With the Music	8126	8224	0.212	0.384	0.66 (1)	0.417
M13 (vs. M7)	Movement Entrainment ~ Arm (vs. Torso) + Half-Time Level (vs. Standard Beat Level) + Double-Time Level (vs. Standard Beat Level) + Piece^1^ + Standing (vs. Sitting) + Urge to Dance + Live Experience	8123	8221	0.221	0.384	4.07 (1)	0.004

Every model includes the random effect (1 | participant). The Akaike information criterion (AIC), Bayesian information criterion (BIC), marginal *R*^2^ (i.e., variance explained by fixed effects only), and conditional *R*^2^ (i.e., variance explained by fixed and random effects) are provided. χ^2^ and *p* values refer to model comparisons to the model by the model stated in brackets using likelihood ratio tests. Null model: Movement entrainment ~ (1 | participant). The best fitting model is marked in bold letters.

^1^Dummy Coding for Pieces 2–8, Piece 1 was used as reference category.

#### Musical factors

To expand the model with *musical factors*, we added the factor *piece* (M2), which showed an improvement of the model fit (compared to M1) (Table 1). The first piece evoked the most movement entrainment compared to the other pieces, for Pieces 2–8: *t*s(3280) = −15.68 to −6.5, *ps* < 0.001 (Supplementary Table S2).

To check the effect of *tempo* instead of *piece*, we replaced *piece* with *tempo* (M3). The model fit was worse than the one of M2 (Table 1), but it showed a significant and positive main effect of *tempo* on movement entrainment. This indicates that pieces with higher tempo evoked more movement entrainment, *t*(3286) = 5.73, *p* < 0.001 (Supplementary Table S3). To further investigate effects of tempo and to check if pieces with tempi in the range of 110–120 BPM (the sweet spot for human motion^13–15^) led to more movement entrainment, we replaced *tempo* with two dummy variables, *slower than sweet spot* (vs. sweet spot) and *faster than sweet spot* (vs. sweet spot) (M4). We used a tolerance range of 5% (pieces with tempo < 104 BPM: coded as *slower than sweet spot*; pieces with tempo > 126 BPM: coded as *faster than sweet spot*). We found a significant and negative main effect for *slower than sweet spot* on movement entrainment, *t*(3285) = −7.80, *p* < 0.001, so there was less movement entrainment during pieces slower than the sweet spot range. We observed no main effect for *faster than sweet spot* (Supplementary Table S4).

As it was likely that participants move more to the half-time level during fast pieces and respectively more to the double-time level during slower pieces, we also tested for interaction between *half-time level* and *faster than sweet spot* as well as *double-time level* and *slower than sweet spot* (M4a). Both interactions were not significant (Supplementary Table S5).

#### Situational factor

As the model fit indices of M3, M4 and M4a were worse than those for M2 (Table 1), we kept *piece* as a *musical factor* and added the concert condition (standing vs. sitting) in a next step as the *situational factor* in our subsequent model (M5), which improved model fit compared to M2 (Table 1). It showed that standing had a positive main effect on movement entrainment compared to sitting, *t*(68) = 4.61, *p* < 0.001 (Supplementary Table S6).

#### Personal factors

Continuing with personal characteristics of the participants, we added all factors of Participatory Dance Experience and Observatory Dance Experience from the Gold-DSI^44^ (M6). As the model fit got worse, we only kept the factor *urge to dance* as it showed the largest absolute *t-*value and smallest *p-*value, *t*(63) = 1.71, *p* = 0.092 (Supplementary Table S7), and computed a new model (M7). The model fit now was improved compared to M5 (Table 1). *Urge to dance* had a significant and positive main effect on movement entrainment, *t*(67) = 2.88, *p* = 0.005 (Supplementary Table S8).

We then added the continuous predictors of *positive activation* (M8), *trait empathy* (M9), *liking the concert* (M10), *liking jazz music* (M11), and *being familiar with the music* (M12) to the model one by one (Supplementary Table S9–13). None of these predictors led to a significant improvement in model fit (Table 1). We also added *live experience* to the model to investigate the effect of the evaluation of experiencing the musicians live (M13). It improved the model fit slightly and significantly (*p* = 0.004). A positive effect of experiencing the musicians live on movement entrainment did not quite reach significance, *t*(66) = 1.99, *p* = 0.051 (Supplementary Table S14).

#### Final model

**Fig. 3:**
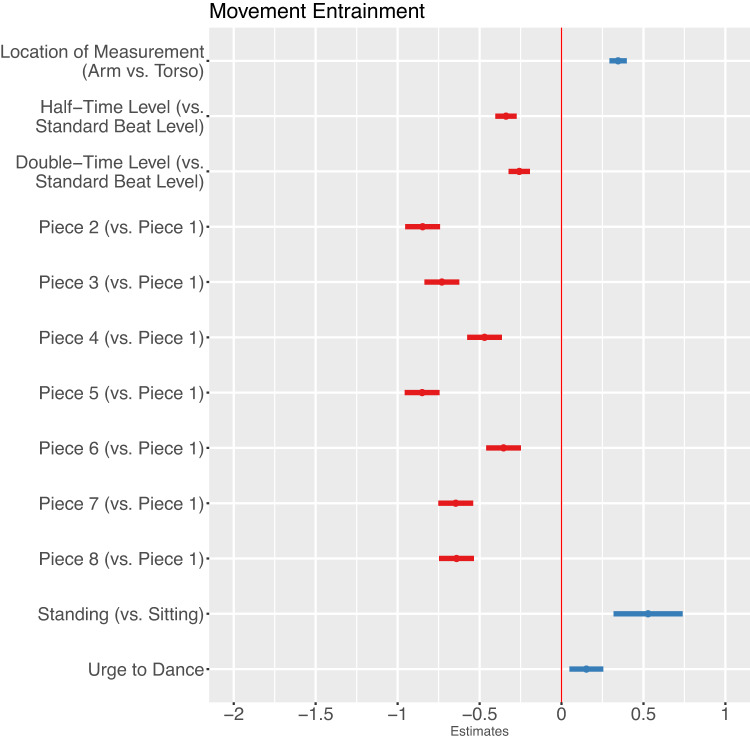
Estimates of the final hierarchical linear model with location of measurement, metrical level, pieces of music, concert situation, and urge to dance as predictors of movement entrainment.

The final model (Fig. 3) shows significant main effects for *factors of measurement*: the location of measurement *arm* with a positive main effect indicates that movement entrainment measured at the arm was higher compared to the torso. Movement entrainment at the *half-time* and *double-time level showed* significant negative main effects, so movement entrainment at the standard beat level was highest compared to the half-time and double-time level. Movement entrainment at the double-time level was relatively higher compared to the half-time level. The different pieces predicted movement entrainment as *musical factors*; movement entrainment during the first piece was the highest compared to the following pieces. As a *situational factor*, the concert condition *standing* showed a positive main effect: there was more movement entrainment in the standing condition compared to the sitting condition. Finally, the *personal factor* of *urge to dance* showed a positive main effect on movement entrainment, indicating a positive relationship of urge to dance and movement entrainment.

## Discussion

We performed two experiments to investigate movement entrainment in ecologically valid concert settings. In the preliminary study, we measured significant movement entrainment to the music and showed that this also was linked to self-reported desire to move and dance to the music. In the main study, we investigated different predictors of movement entrainment and identified significant effects of *factors of measurement*, *musical factors*, a *situational factor*, and a *personal factor*.

In addition to studies that measured movement in laboratory or artificial settings^29–32^, as well as ecologically valid concert settings^33–35^, our study is the first to measure movement entrainment in a concert, where participants were free to move, even dance, at least in the standing condition. With the small devices, participants could behave as they would usually do in a concert. This expands the methods and possibilities of concert research and strongly implies that it is possible to conduct concert research in ecologically valid settings (RQ_1_).

Concerning *factors of measurement* as predictors of movement entrainment, we found an effect of the location of measurement in our main study. The measurement at the arm revealed more movement entrainment than at the torso. It is conceivable that this finding is linked to the complexity of arm movements as they can move independently of other parts of the body and in different directions^26^.

In terms of the metrical level, people tended to move most on the standard beat level, followed by the double-time and the half-time levels. This suggests that overall, people mostly moved to the beat at the piece’s tempo (82 to 156 BPM). We also found that the tempo of the music was positively associated with movement entrainment: Faster tempi led to more movement entrainment. Testing the effects of tempi outside the ‘sweet spot’ range of 110–120 BPM showed that participants entrained less to slower pieces; there was no effect for faster pieces. These results are partly consistent with those of Burger et al.^17^, who did not find an effect of tempo on movement features. Furthermore, we found no evidence that tempi slower or faster than the ‘sweet spot’ range led to more movement entrainment at the double-time or half-time levels, respectively. To explain this, we must consider that the tempo range of the pieces was moderate. There were no pieces with extremely slow or fast tempi, so it wasn’t necessary to entrain on other metrical levels.

We also identified an effect of the pieces as a *musical factor*: Movement entrainment was highest during the first piece *In the Stone* compared to the other ones. This is not surprising considering the song’s description as an “irresistible dancing cut” in the contemporary music magazine Cash Box^4^. Perhaps the other pieces were simply less danceable than the opening piece, and therefore evoked less movement entrainment.

However, the factor *piece* showed the best contribution to improving model fit in our model compared to tempo or the tempo sweet spot. This can be explained by the fact that *piece* contains much more information than just the tempo and therefore it is the most suitable *musical factor* we tested to explain movement entrainment. A differentiated approach, in which other musical parameters are considered and included in a more sophisticated way, also in the context of groove research^49^, would be a potential direction for further investigation. Nevertheless, this would extend beyond the focus of the present paper.

In the context of *situational factors*, it is evident that individuals may exhibit more movement entrainment at a standing concert compared to one where they are seated. Our study provides empirical support for this assumption: Participants in the standing condition showed significantly higher levels of movement entrainment than those in the seated condition. Sitting in a chair during a concert restricts physical movement and may therefore inhibit movement entrainment. This could alter the overall concert experience as a result.

We expected several *personal factors* to have an influence on movement entrainment. Concerning dance sophistication^44^, only *urge to dance* was significant. This indicates that people who generally feel an urge to dance to music also tend to entrain more in a concert. Our findings further suggest that the other factors of participatory and observatory dance sophistication are not that strongly linked to movement entrainment.

Finally, we found preliminary support for the conclusion that the positive evaluation of experiencing the musicians live has a positive effect on movement entrainment. It could be that the unique situation of experiencing the musicians and their playing in a live concert makes people more involved in the music, leading to more movement entrainment (RQ_2_).

Although previous research suggests a connection between self-reported positive affect and faster and more complex movements while dancing to music^25^, we could not find an association of positive activation and movement entrainment in our study. A reason for this finding might be that moving fast or with complex movements to music must be distinguished from movement entrainment. Therefore, we can conclude that positive mood does not seem to be automatically associated with movement entrainment.

Moreover, in contrast to Bamford and Davidson^45^, we could not find a positive association between trait empathy and movement entrainment. One possible explanation for this is that our measure of empathy was shorter, consisting of only four items. This helped to keep the questionnaire as short as possible and made participation in the study more pleasant. Another reason why our findings might contradict to the cited study is that we conducted our study in the field and not in the laboratory. In a situation with higher ecological validity the effect could be weaker, and it is possible that we could not detect it due to a lack of statistical power.

Additionally, we could not find a positive association between being familiar with the music and movement entrainment. This contrasts with the findings of a previous study^46^ whose authors report associations between participants’ movement in a silent disco and their familiarity with the popular pop and party songs. One possible explanation is that our measure of familiarity was taken after the concert based on all the musical excerpts to avoid disturbing individuals’ concert experience. Therefore, it is possible that if we had measured the familiarity of each piece during the concert, a relationship might still have been found. Another possible explanation why we could not find this association might be the specific genre of music we examined. Usually, big bands play arrangements of famous jazz standards or other popular pieces. That was also the case in our experiment. It is likely that participants who stated to be familiar with the music in our study were familiar with the pieces played, but probably not with the specific arrangements. The band played an arrangement of The Beatles’ *Elanor Rigby*, for example. It is likely that many of the participants knew this song and other songs played at the concert and reported this as familiarity in the questionnaire. However, they were probably unfamiliar with the specific arrangements, which may have resulted in less movement entrainment. Nevertheless, even in a concert where participants were fans of the performer but were unfamiliar with the songs, they still entrained more to the music than neutral listeners^33^ which suggests that familiarity with the specific pieces might not be necessary to evoke entrained movement. We have also found no indication that liking jazz music or liking the concert is associated with movement entrainment. Concerning liking the music, it is reasonable that liking jazz music is not comparable in intensity and involvement to being a fan of a specific band. Also, the term jazz music unites a wide variety of music and might be not precise enough to describe big band music. Regarding liking the concert, the lack of variance in the item *liking the concert* (90% of the participants rated 4 or 5 on a 5-point scale) suggests that this variable was influenced by a ceiling effect. This could explain why it was not possible to observe a significant effect of liking the concert on movement entrainment. More work is needed to understand how familiarity and preference affect movement entrainment.

The present study represents a first attempt to explore movement entrainment in an ecologically valid live concert setting. There are several limitations to be mentioned here. First, due to the accelerometer devices used in this study, we can only make a statement about peoples’ movement to the music. As people were allowed to move freely in the room, we did not actually know where they were in the room and if they danced with each other. There are more accurate methods such as motion capture with multiple body markers^29,30^ or only a few attached to the head^33^ that would make it possible to also measure participants’ location in a room. However, using motion capture in a concert hall with many people moving around requires a very sophisticated technical setup and possibly more restrictions for participants. Additionally, we could not control for what participants did during the concert while we collected their acceleration data. There were no instructions given on how to behave during the concert. This resulted in some participants holding bottles or mobile phones in their hands on the side to which the measuring device was attached. A trade-off exists between quality and accuracy of the data and the non-invasiveness of such recordings. The objective of this study was to focus solely on ecological validity, so we periodized the unrestricted concert experience of our participants over the accuracy of our data. We argue that the possibility to measure a relatively large sample compensates for the fact that other methods of measurement would be more precise.

It is also important to acknowledge that some of our findings are not generalizable beyond the specific style of music and type of concerts we studied. The finding that people mostly moved to the standard beat level and rarely to the double-time and half-time level, and that tempo was positively associated with movement entrainment, is very likely depended on the average and usual tempi of the pieces played during the concert. Concerts with different musical styles and different tempi conventions might lead to other findings here. There are many different concert cultures, and much work remains to be done for a full understanding of movement entrainment during live concerts and its relation to concert experience.

Despite these limitations, the present study has shown a suitable method to measure movement entrainment in an ecologically valid concert setting. Future research should concentrate on different musical genres to be able to make general statements. Due to the lack of variance in the measurement of liking the concert, it could be appropriate to conduct a concert with a more diverse audience, including fans and non-fans, for example^33^. In light of the theory of the concert frame^40^, future research could also focus more differentiated on the effects of listeners’ personality on movement entrainment, as well as on different concert formats. Our research can be seen as a first step towards exploring movement entrainment in ecologically valid concert settings.

## Methods

Participants of both studies were informed about the study procedure in advance and gave their written consent. The study was approved by the ethics committee of the DGM (the German Association for Music Psychology) and was performed in accordance with their guidelines and regulations. These also refer to the regulations of the DGPs (the German Psychological Society).

### Preliminary study

#### Participants

We collected data from 42 volunteers (female = 24, male = 17, other = 1; mean age: 26.2 years, *SD* = 9.8) recruited through email, social media, posters, and in person from attendees at the free concert, immediately before it started. Participants were mainly students (81%) and 69% stated in the questionnaire that they describe themselves as musicians. 64% of the participants came to the concert with someone else. At the end of the experiment, all participants received 10 Euro compensation in cash. We excluded one participant’s data for the first eleven pieces and a second participant’s data for the last 15 pieces because they were part of the organizing committee for the student concert and helped with tasks such as selling drinks.

#### Stimuli

The experiment was conducted during a concert at the Music Institute of TU Dortmund University. The first part of the concert featured six solo pop songs, performed by a number of students. The second part featured four different rock bands, each performing three to four cover songs. The last part of the concert was a brass band playing 13 pieces, mostly cover versions of popular pieces from the pop genre (for the set list with BPM values, see Supplementary Table S15). The concert started at 7:15 PM and ended at around 10 PM on a Wednesday evening. It took place in a concert room in the basement of a university building which is provided with a stage, sound system, and stage lights. There were in total around 100 people attending the concert, drinks were provided and everyone, including the participants, was allowed to enter and leave the concert hall at any time.

#### Movement data

Movement data for each participant were collected using the built-in accelerometer of two wearable devices (28 g and 31 g, each 65 × 32 × 12 mm). The Shimmer GSR + sensor was attached to the participants’ non-dominant arm wrists and also measured skin conductance at the index finger and middle finger; the Shimmer ECG sensor was attached with a band around the participants’ torso and also measured their electrocardiogram with two electrodes placed at the participants’ chest. Both devices measured acceleration in two ways: a 3-axis low noise accelerometer and a 3-axis wide range accelerometer. We decided to use both accelerometers because we expected a wide range of motion. By this, we could both measure large accelerations as well as smaller movements accurately.

Data was recorded on each device’s SD card (Sampling rate GSR + device: 128 Hz; ECG device: 256 Hz). Please note that the data of skin conductance and the electrocardiogram are not used in the analysis of this article. All movement data was recorded with a real-world timestamp from a Windows PC laptop that was running Shimmer’s software ConsensysPro. To determine the exact time of the beginning of the concert and to be able to synchronize the acceleration data with the audio-visual recordings, we took additional recordings of the laptop screen showing its real-world time with the surrounding sound from the concert hall.

#### Pre-concert questionnaire

Before the concert, we asked participants for sociodemographic data, if they were students, and if they attended the concert alone or in company.

#### Post-concert questionnaire

In the post-concert questionnaire, we asked for the participants’ concert experience and if they had the desire to move and dance on a 5-point scale (1 = *not at all*, 5 = *very much*).

#### Audio-visual recordings

The concert was recorded with two cameras. One facing the audience and one facing the performers on the stage during the entire duration of the concert.

#### Procedure

Participants were greeted at the entrance, received an information sheet about the experiment, and filled out the consent form. They then filled out the pre-concert questionnaire at tables provided in the foyer of the concert room. This took about ten minutes. To be able to assign the questionnaires to the movement data, we used anonymous IDs generated by the participants themselves. Then participants were fitted with the two devices. Participants were not restricted in their movement by the devices worn on their bodies. After the measuring devices were attached, the participants were allowed to move freely in the building. They were not given any further instructions, and they were not encouraged to behave or move in any particular way during the concert. After the concert, participants filled in the post-concert questionnaire. This took about ten minutes. After completing the questionnaire, the participants were thanked and received their compensation for the participation.

Participants were further allowed to leave the concert at any time they wanted and to finish the experiment before it ended. There were no more COVID restrictions, yet some people voluntarily wore masks during the event. After the experiment, participants were debriefed.

#### Data analysis

##### Musical feature extraction

The tempi of the played pieces at the concert were determined by two musically trained raters who listened to the recording of the concert and tapped along to the beat of each played piece using a metronome application. The mirtempo function of the MIR toolbox^50^ was used to confirm the tempo analysis. Due to very unclear tempi, we had to exclude four pieces from the analysis.

##### Movement entrainment

We defined the degree of movement entrainment of each participant as the extent to which they moved their body with the frequency of the beat of each piece played at the concert.

For every participant, we had two data sets: One from the device at the arm and one from the device at the torso. Each data set consisted of six acceleration values: three values for the three dimensions of the low noise accelerometer and three values for the three dimensions of the wide range accelerometer.

Using MATLAB (Mathworks, Version R2024b), we first conducted an interpolation, using MATLAB function interp1. It is applied to each signal to assign the same number of temporal data points between two reference points. Then the full concert was segmented into the pieces of music.

Since the sensors measured acceleration on three axes, we calculated the square root of the sum of the squares of the three axes x, y and z as a measure of the overall magnitude of movement ($\:\sqrt{{x}^{2}+{y}^{2}+{z}^{2}}$). We used the mean of the low noise and the wide range accelerometer data, as the data was visually very similar, only slightly shifted up or down (see Suppelementary Material S17–S19 for raw data and step-by-step plots for the analysis). We then applied a fast Fourier transform, using MATLAB function fft, to determine the extent to which the participants were moving at each frequency. Next, we extracted the power of the movement frequency within the range of the three metrical levels of the tempo of each piece: half the beat period (0.5 BPM; half-time), the standard beat level (BPM), and two times the beat period (2 BPM; double-time) within a tolerance range of ± 5%^21^. Therefore, there were six values of entrainment for each of the 34 pieces for every participant: 2 devices × 3 different metrical levels.

### Main study

#### Participants

A total of 70 participants attended one of the two concerts. Due to incomplete data sets we had to exclude one participant, resulting in a final sample of 69 participants. Of these, 28 participants (female = 13, male = 15; mean age: 30.1 years, *SD* = 10.4) attended Concert 1, and 41 participants (female = 21, male = 18, other = 1, 1 preferred not to say; mean age: 28.5, *SD* = 11.5) attended Concert 2. We used a short version with three items per subscale of the German version of the Goldsmiths Musical Sophistication Index^51,52^ to assess the participants’ general musical sophistication (Concert 1: *M* = 15.1, *SD* = 5.3; Concert 2: *M =* 14.5, *SD* = 5.4). Most participants in both concerts indicated that their highest educational level was at least a university degree (Concert 1: 64.3%; Concert 2: 48.8%) or that they were still in vocational training, e.g., trainee, student, or apprentice (Concert 1: 25.0%; Concert 2: 34.1%). Most of the participants (Concert 1: 82.1%; Concert 2: 73.2%) came to the concert with someone else. They were recruited via email, social media, posters, word-of-mouth, and street recruitment in front of the university canteen. The concert was free of charge and participants were offered a free drink after they checked in to participate in the study. Each participant listened to only one of the concerts, the one with the sitting condition or the one with the standing condition. Participants did not know beforehand, which concert condition (sitting or standing) they were attending or that this was part of the experiment at all.

#### Stimuli

The experiment was conducted during two big band concerts on two Wednesday evenings in two consecutive weeks in the concert hall of TU Dortmund University. The band consisted of 18 musicians. At both concerts, the band played the same big band arrangements of seven pieces (for the set list with BPM values, see Supplementary Table S16). The last piece was played two times: The first time, the singer explained the piece and taught the audience how to sing along; afterwards, the band started from the beginning and played the piece to the end. The band was instructed to play as similarly as possible on both evenings. The concert started at around 7:50 PM both times, with a delay of ca. 20 min. The reasons for the delay were different at each concert: In week one, the conductor of the band arrived late because of a traffic jam; in week two, many participants arrived very close to the start of the concert and attaching the measuring devices took too long. The concerts lasted around 50 min each.

#### Movement data

The measurement of movement data was the same as in the preliminary study.

#### Pre-concert questionnaire

Before the concert, participants reported their preference of jazz music and several other musical genres (using the genre categories from the STOMP-R^53^). To measure empathy, we used the German version^54^ of the Interpersonal Reactivity Index^55^. To keep the questionnaire as short as possible, we used a short version which consists of two items for each of the dimensions *perspective taking* and *empathic concern*. The items were answered on a 5-point Likert scale (1 = *doesn’t apply at all*, 5 = *applies completely*). To assess dance sophistication, we shortened the Dance Sophistication Index which measures both participatory (P) and observational (O) dance experience^44^, and only used the item with the highest loading for each of the five factors *body awareness* (P1.4), *social dancing* (P2.3), *urge to dance* (P3.3), *dance training* (P4.3), and *observational dance experience* (O1.2). These items were answered on a 7-point scale (1 = *completely agree*, 7 = *completely disagree*). We further asked for sociodemographic data and if participants attended the concert alone or accompanied.

#### Post-concert questionnaire

After the concert, we measured affective states using the ’Positive Activation Negative Activation Valence’ scale, short version (PANAVA-KS)^56^. The scale comprises ten bipolar items on a seven-point Likert scale and forms three subscales: *positive activation* (PA), *negative activation* (NA), and *valence* (VA). We asked further questions about their concert experience, if they had the desire to move and dance during the concert on a 5-point scale (1 = *not at all*, 5 = *very much*), their familiarity with the heard music (on a scale from 1 = *I knew all pieces*, to 3 = *I didn’t know a single piece*), how they liked the concert on a 5-point scale (1 = *very bad*, 5 = *very good*), and how they felt about experiencing the musicians live on a rating scale (1 = *very bad*, to 5 = *very good*). We also asked the participants if they had any impairments due to alcohol or other drugs, or if they had any hearing impairments. We also asked if they had physical limitations that affected their ability to sit or stand.

#### Audio-visual recordings

Analogous to the preliminary study, the concerts were recorded with two cameras. One camera filmed the audience; the other camera filmed the band on the stage.

#### Procedure

The procedure was the same as in the preliminary study, with the exception that participants did not receive a financial compensation for their participation (except from the free drink). Concert 1 was seated; Concert 2 took place in the same room but was unseated and the audience had to stand during the concert.

#### Data analysis

##### Musical feature extraction and analysis of movement entrainment

The musical feature extraction and the analysis of movement entrainment followed the one described for the preliminary study.

##### Statistical analysis

For analyzing data we used IBM SPSS Statistics, JASP^57^ and the lme4 package^47^ in R^48^. We fitted several Hierarchical Linear Models following a forward fitting approach to explain movement entrainment. Missing values were replaced by mean replacement as a maximum of four values were missing in each variable. We tested the following fixed effects: Factors of measurement, musical factors, situational factor, and personal factors. All continuous variables were z-standardized. The random effect, coded as (1 | participant), accounted for individual differences by allowing a random intercept per participant. The null-model only included the random effect. Afterwards, we included the fixed effects step-by-step. We compared the fit of the models using likelihood-ratio tests.

## Supplementary Information

Below is the link to the electronic supplementary material.

Supplementary Material 1

## Data Availability

The MATLAB and R code for the analysis are available on the Open Science Framework repository: osf.io/rw9nh.
