# Supplementary material for: Exploring movement entrainment in an ecologically valid concert setting
Source: Sci Rep. 2025 Aug 29;15:31796. doi: 10.1038/s41598-025-13376-7 (PMC12394413; doi:10.1038/s41598-025-13376-7)
Supplement: Supplementary file 1 — Supplementary Material 1 [file 41598_2025_13376_MOESM1_ESM.pdf]

# Supplementary Material

# Exploring movement entrainment in an ecologically valid concert setting

Maren Hochgesand[1] & Hauke Egermann[1]

[1]Cologne Systematic Musicology Lab, Institute of Musicology, University of Cologne, Cologne, Germany

Corresponding author:
Prof. Dr. Hauke Egermann
Cologne Systematic Musicology Lab
Institute of Musicology
University of Cologne
Albertus-Magnus-Platz
D-50923 Cologne
Germany

Email: hauke.egermann@uni-koeln.de

**Table S1.** Overview of M1. HLM with movement entrainment as the dependent variable.

| M1 | | | | | |
|---|---|---|---|---|---|
| Value | Estimate | *SE* | *df* | *t* | *p* |
| (Intercept) | 0.03 | 0.07 | 3287 | 0.39 | 0.699 |
| Arm (vs. Torso) | 0.35 | 0.03 | 3287 | 12.04 | <0.001 |
| Half Time Level (vs. BPM Level) | -0.34 | 0.04 | 3287 | -9.65 | <0.001 |
| Double Time Level (vs. BPM Level) | -0.26 | 0.04 | 3287 | -7.34 | <0.001 |

**Table S2.** Overview of M2. HLM with movement entrainment as the dependent variable.

| M2 | | | | | |
|---|---|---|---|---|---|
| Value | Estimate | *SE* | *df* | *t* | *p* |
| (Intercept) | 0.59 | 0.08 | 3280 | 7.78 | <0.001 |
| Arm (vs. Torso) | 0.35 | 0.03 | 3280 | 12.73 | <0.001 |
| Half Time Level (vs. BPM Level) | -0.34 | 0.03 | 3280 | -10.19 | <0.001 |
| Double Time Level (vs. BPM Level) | -0.26 | 0.03 | 3280 | -7.76 | <0.001 |
| Piece 2 (vs. Piece 1) | -0.85 | 0.05 | 3280 | -15.62 | <0.001 |
| Piece 3 (vs. Piece 1) | -0.73 | 0.05 | 3280 | -13.46 | <0.001 |
| Piece 4 (vs. Piece 1) | -0.47 | 0.05 | 3280 | -8.66 | <0.001 |
| Piece 5 (vs. Piece 1) | -0.85 | 0.05 | 3280 | -15.68 | <0.001 |
| Piece 6 (vs. Piece 1) | -0.35 | 0.05 | 3280 | -6.52 | <0.001 |
| Piece 7 (vs. Piece 1) | -0.65 | 0.05 | 3280 | -11.90 | <0.001 |
| Piece 8 (vs. Piece 1) | -0.64 | 0.05 | 3280 | -11.82 | <0.001 |

**Table S3.** Overview of M3. HLM with movement entrainment as the dependent variable.

| M3 | | | | | |
|---|---|---|---|---|---|
| Value | Estimate | *SE* | *df* | *t* | *p* |
| (Intercept) | -0.31 | 0.08 | 3286 | -4.04 | <0.001 |
| Arm (vs. Torso) | 0.35 | 0.03 | 3286 | 12.10 | <0.001 |
| Half Time Level (vs. BPM Level) | -0.34 | 0.03 | 3286 | -9.70 | <0.001 |
| Double Time Level (vs. BPM Level) | -0.26 | 0.03 | 3286 | -7.38 | <0.001 |
| Tempo | 0.08 | 0.01 | 3286 | 5.73 | <0.001 |

**Table S4.** Overview of M4. HLM with movement entrainment as the dependent variable.

| M4 | | | | | |
|---|---|---|---|---|---|
| Value | Estimate | *SE* | *df* | *t* | *p* |
| (Intercept) | 0.09 | 0.07 | 3285 | 1.22 | 0.221 |
| Arm (vs. Torso) | 0.35 | 0.03 | 3285 | 12.19 | <0.001 |
| Half Time Level (vs. BPM Level) | -0.34 | 0.03 | 3285 | -9.76 | <0.001 |
| Double Time Level (vs. BPM Level) | -0.26 | 0.03 | 3285 | -7.43 | <0.001 |
| Slower than Sweet Spot (vs. Sweet Spot) | -0.30 | 0.04 | 3285 | -7.80 | <0.001 |
| Faster than Sweet Spot (vs. Sweet Spot) | 0.03 | 0.03 | 3285 | 0.77 | 0.440 |

**Table S5.** Overview of M4a. HLM with movement entrainment as the dependent variable.

| M4a | | | | | |
|---|---|---|---|---|---|
| Value | Estimate | *SE* | *df* | *t* | *p* |
| (Intercept) | 0.08 | 0.07 | 3283 | 1.16 | 0.247 |
| Arm (vs. Torso) | 0.35 | 0.03 | 3283 | 12.20 | <0.001 |
| Half Time Level (vs. BPM Level) | -0.30 | 0.04 | 3283 | -7.51 | <0.001 |
| Faster than Sweet Spot (vs. Sweet Spot) | 0.069 | 0.04 | 3283 | 1.69 | 0.0917 |
| Double Time Level (vs. BPM Level) | -0.28 | 0.04 | 3283 | -7.39 | <0.001 |
| Slower than Sweet Spot (vs. Sweet Spot) | -0.34 | 0.05 | 3283 | -7.44 | <0.001 |
| Half Time Level (vs. BPM Level) × Faster than Sweet Spot (vs. Sweet Spot) | 0.12 | 0.07 | 3283 | 1.56 | 0.119 |
| Double Time Level (vs. BPM Level) × Slower than Sweet Spot (vs. Sweet Spot) | -0.13 | 0.07 | 3283 | -1.91 | 0.057 |

**Table S6.** Overview of M5. HLM with movement entrainment as the dependent variable.

| M5 | | | | | |
|---|---|---|---|---|---|
| Value | Estimate | *SE* | *df* | *t* | *p* |
| (Intercept) | 0.28 | 0.10 | 3280 | 2.88 | 0.004 |
| Arm (vs. Torso) | 0.35 | 0.03 | 3280 | 12.73 | <0.001 |
| Half Time Level (vs. BPM Level) | -0.34 | 0.03 | 3280 | -10.19 | <0.001 |
| Double Time Level (vs. BPM Level) | -0.26 | 0.03 | 3280 | -7.76 | <0.001 |
| Piece 2 (vs. Piece 1) | -0.85 | 0.05 | 3280 | -15.62 | <0.001 |
| Piece 3 (vs. Piece 1) | -0.73 | 0.05 | 3280 | -13.46 | <0.001 |
| Piece 4 (vs. Piece 1) | -0.47 | 0.05 | 3280 | -8.66 | <0.001 |
| Piece 5 (vs. Piece 1) | -0.85 | 0.05 | 3280 | -15.68 | <0.001 |
| Piece 6 (vs. Piece 1) | -0.35 | 0.05 | 3280 | -6.52 | <0.001 |
| Piece 7 (vs. Piece 1) | -0.65 | 0.05 | 3280 | -11.90 | <0.001 |
| Piece 8 (vs. Piece 1) | -0.64 | 0.05 | 3280 | -11.81 | <0.001 |
| Standing (vs. Sitting) | 0.52 | 0.11 | 68 | 4.61 | <0.001 |

**Table S7.** Overview of M6. HLM with movement entrainment as the dependent variable.

| M6 | | | | | |
|---|---|---|---|---|---|
| Value | Estimate | *SE* | *df* | *t* | *p* |
| (Intercept) | 0.29 | 0.10 | 3280 | 2.96 | 0.0031 |
| Arm (vs. Torso) | 0.35 | 0.03 | 3280 | 12.73 | <0.001 |
| Half Time Level (vs. BPM Level) | -0.34 | 0.03 | 3280 | -10.19 | <0.001 |
| Double Time Level (vs. BPM Level) | -0.26 | 0.03 | 3280 | -7.76 | <0.001 |
| Piece 2 (vs. Piece 1) | -0.85 | 0.05 | 3280 | -15.62 | <0.001 |
| Piece 3 (vs. Piece 1) | -0.73 | 0.05 | 3280 | -13.46 | <0.001 |
| Piece 4 (vs. Piece 1) | -0.47 | 0.05 | 3280 | -8.66 | <0.001 |
| Piece 5 (vs. Piece 1) | -0.85 | 0.05 | 3280 | -15.68 | <0.001 |
| Piece 6 (vs. Piece 1) | -0.35 | 0.05 | 3280 | -6.52 | <0.001 |
| Piece 7 (vs. Piece 1) | -0.65 | 0.05 | 3280 | -11.90 | <0.001 |
| Piece 8 (vs. Piece 1) | -0.64 | 0.05 | 3280 | -11.81 | <0.001 |
| Standing (vs. Sitting) | 0.51 | 0.12 | 63 | 4.34 | <0.001 |
| Urge to Dance | 0.13 | 0.07 | 63 | 1.71 | 0.092 |
| Body Awareness | -0.03 | 0.06 | 63 | -0.48 | 0.632 |
| Dance Training | -0.02 | 0.06 | 63 | -0.29 | 0.770 |
| Observational Dance Experience | 0.01 | 0.06 | 63 | 0.19 | 0.848 |
| Social Dancing | 0.04 | 0.07 | 63 | 0.58 | 0.565 |

**Table S8.** Overview of M7. HLM with movement entrainment as the dependent variable.

| M7. | | | | | |
|---|---|---|---|---|---|
| Value | Estimate | *SE* | *df* | *t* | *p* |
| (Intercept) | 0.28 | 0.09 | 3280 | 2.95 | 0.003 |
| Arm (vs. Torso) | 0.35 | 0.03 | 3280 | 12.73 | <0.001 |
| Half Time Level (vs. BPM Level) | -0.34 | 0.03 | 3280 | -10.19 | <0.001 |
| Double Time Level (vs. BPM Level) | -0.26 | 0.03 | 3280 | -7.76 | <0.001 |
| Piece 2 (vs. Piece 1) | -0.85 | 0.05 | 3280 | -15.62 | <0.001 |
| Piece 3 (vs. Piece 1) | -0.73 | 0.05 | 3280 | -13.46 | <0.001 |
| Piece 4 (vs. Piece 1) | -0.47 | 0.05 | 3280 | -8.66 | <0.001 |
| Piece 5 (vs. Piece 1) | -0.85 | 0.05 | 3280 | -15.68 | <0.001 |
| Piece 6 (vs. Piece 1) | -0.35 | 0.05 | 3280 | -6.52 | <0.001 |
| Piece 7 (vs. Piece 1) | -0.65 | 0.05 | 3280 | -11.90 | <0.001 |
| Piece 8 (vs. Piece 1) | -0.64 | 0.05 | 3280 | -11.81 | <0.001 |
| Standing (vs. Sitting) | 0.53 | 0.11 | 67 | 4.91 | <0.001 |
| Urge to Dance | 0.15 | 0.05 | 67 | 2.88 | 0.005 |

**Table S9.** Overview of M8. HLM with movement entrainment as the dependent variable.

| M8 | | | | | |
|---|---|---|---|---|---|
| Value | Estimate | *SE* | *df* | *t* | *p* |
| (Intercept) | 0.29 | 0.09 | 3280 | 3.09 | 0.002 |
| Arm (vs. Torso) | 0.35 | 0.03 | 3280 | 12.73 | <0.001 |
| Half Time Level (vs. BPM Level) | -0.34 | 0.03 | 3280 | -10.19 | <0.001 |
| Double Time Level (vs. BPM Level) | -0.26 | 0.03 | 3280 | -7.76 | <0.001 |
| Piece 2 (vs. Piece 1) | -0.85 | 0.05 | 3280 | -15.62 | <0.001 |
| Piece 3 (vs. Piece 1) | -0.73 | 0.05 | 3280 | -13.46 | <0.001 |
| Piece 4 (vs. Piece 1) | -0.47 | 0.05 | 3280 | -8.66 | <0.001 |
| Piece 5 (vs. Piece 1) | -0.85 | 0.05 | 3280 | -15.68 | <0.001 |
| Piece 6 (vs. Piece 1) | -0.35 | 0.05 | 3280 | -6.52 | <0.001 |
| Piece 7 (vs. Piece 1) | -0.65 | 0.05 | 3280 | -11.90 | <0.001 |
| Piece 8 (vs. Piece 1) | -0.64 | 0.05 | 3280 | -11.81 | <0.001 |
| Standing (vs. Sitting) | 0.50 | 0.11 | 66 | 4.60 | <0.001 |
| Urge to Dance | 0.17 | 0.06 | 66 | 3.11 | 0.003 |
| Positive Activation | -0.07 | 0.06 | 66 | -1.18 | 0.244 |

**Table S10.** Overview of M9. HLM with movement entrainment as the dependent variable.

| M9 | | | | | |
|---|---|---|---|---|---|
| Value | Estimate | *SE* | *df* | *t* | *p* |
| (Intercept) | 0.28 | 0.09 | 3280 | 2.99 | 0.003 |
| Arm (vs. Torso) | 0.35 | 0.03 | 3280 | 12.73 | <0.001 |
| Half Time Level (vs. BPM Level) | -0.34 | 0.03 | 3280 | -10.19 | <0.001 |
| Double Time Level (vs. BPM Level) | -0.26 | 0.03 | 3280 | -7.76 | <0.001 |
| Piece 2 (vs. Piece 1) | -0.85 | 0.05 | 3280 | -15.62 | <0.001 |
| Piece 3 (vs. Piece 1) | -0.73 | 0.05 | 3280 | -13.46 | <0.001 |
| Piece 4 (vs. Piece 1) | -0.47 | 0.05 | 3280 | -8.66 | <0.001 |
| Piece 5 (vs. Piece 1) | -0.85 | 0.05 | 3280 | -15.68 | <0.001 |
| Piece 6 (vs. Piece 1) | -0.35 | 0.05 | 3280 | -6.52 | <0.001 |
| Piece 7 (vs. Piece 1) | -0.65 | 0.05 | 3280 | -11.90 | <0.001 |
| Piece 8 (vs. Piece 1) | -0.64 | 0.05 | 3280 | -11.81 | <0.001 |
| Standing (vs. Sitting) | 0.52 | 0.11 | 66 | 4.82 | <0.001 |
| Urge to Dance | 0.13 | 0.06 | 66 | 2.37 | 0.021 |
| Trait Empathy | 0.05 | 0.06 | 66 | 0.94 | 0.349 |

**Table S11.** Overview of M10. HLM with movement entrainment as the dependent variable.

| M10 | | | | | |
|---|---|---|---|---|---|
| Value | Estimate | *SE* | *df* | *t* | *p* |
| (Intercept) | 0.28 | 0.09 | 3280 | 2.91 | 0.004 |
| Arm (vs. Torso) | 0.35 | 0.03 | 3280 | 12.73 | <0.001 |
| Half Time Level (vs. BPM Level) | -0.34 | 0.03 | 3280 | -10.19 | <0.001 |
| Double Time Level (vs. BPM Level) | -0.26 | 0.03 | 3280 | -7.76 | <0.001 |
| Piece 2 (vs. Piece 1) | -0.85 | 0.05 | 3280 | -15.62 | <0.001 |
| Piece 3 (vs. Piece 1) | -0.73 | 0.05 | 3280 | -13.46 | <0.001 |
| Piece 4 (vs. Piece 1) | -0.47 | 0.05 | 3280 | -8.66 | <0.001 |
| Piece 5 (vs. Piece 1) | -0.85 | 0.05 | 3280 | -15.68 | <0.001 |
| Piece 6 (vs. Piece 1) | -0.35 | 0.05 | 3280 | -6.52 | <0.001 |
| Piece 7 (vs. Piece 1) | -0.65 | 0.05 | 3280 | -11.90 | <0.001 |
| Piece 8 (vs. Piece 1) | -0.64 | 0.05 | 3280 | -11.81 | <0.001 |
| Standing (vs. Sitting) | 0.53 | 0.11 | 66 | 4.88 | <0.001 |
| Urge to Dance | 0.15 | 0.05 | 66 | 2.77 | 0.007 |
| Liking the Concert | 0.02 | 0.05 | 66 | 0.29 | 0.774 |

**Table S12.** Overview of M11. HLM with movement entrainment as the dependent variable.

| M11 | | | | | |
|---|---|---|---|---|---|
| Value | Estimate | *SE* | *df* | *t* | *p* |
| (Intercept) | 0.28 | 0.09 | 3280 | 2.95 | 0.003 |
| Arm (vs. Torso) | 0.35 | 0.03 | 3280 | 12.73 | <0.001 |
| Half Time Level (vs. BPM Level) | -0.34 | 0.03 | 3280 | -10.19 | <0.001 |
| Double Time Level (vs. BPM Level) | -0.26 | 0.03 | 3280 | -7.76 | <0.001 |
| Piece 2 (vs. Piece 1) | -0.85 | 0.05 | 3280 | -15.62 | <0.001 |
| Piece 3 (vs. Piece 1) | -0.73 | 0.05 | 3280 | -13.46 | <0.001 |
| Piece 4 (vs. Piece 1) | -0.47 | 0.05 | 3280 | -8.66 | <0.001 |
| Piece 5 (vs. Piece 1) | -0.85 | 0.05 | 3280 | -15.68 | <0.001 |
| Piece 6 (vs. Piece 1) | -0.35 | 0.05 | 3280 | -6.52 | <0.001 |
| Piece 7 (vs. Piece 1) | -0.65 | 0.05 | 3280 | -11.90 | <0.001 |
| Piece 8 (vs. Piece 1) | -0.64 | 0.05 | 3280 | -11.81 | <0.001 |
| Standing (vs. Sitting) | 0.53 | 0.11 | 66 | 4.87 | <0.001 |
| Urge to Dance | 0.15 | 0.05 | 66 | 2.83 | 0.006 |
| Liking Jazz Music | 0.04 | 0.05 | 66 | 0.67 | 0.502 |

**Table S13.** Overview of M12. HLM with movement entrainment as the dependent variable.

| M12 | | | | | |
|---|---|---|---|---|---|
| Value | Estimate | *SE* | *df* | *t* | *p* |
| (Intercept) | 0.28 | 0.09 | 3280 | 2.95 | 0.003 |
| Arm (vs. Torso) | 0.35 | 0.03 | 3280 | 12.73 | <0.001 |
| Half Time Level (vs. BPM Level) | -0.34 | 0.03 | 3280 | -10.19 | <0.001 |
| Double Time Level (vs. BPM Level) | -0.26 | 0.03 | 3280 | -7.76 | <0.001 |
| Piece 2 (vs. Piece 1) | -0.85 | 0.05 | 3280 | -15.62 | <0.001 |
| Piece 3 (vs. Piece 1) | -0.73 | 0.05 | 3280 | -13.46 | <0.001 |
| Piece 4 (vs. Piece 1) | -0.47 | 0.05 | 3280 | -8.66 | <0.001 |
| Piece 5 (vs. Piece 1) | -0.85 | 0.05 | 3280 | -15.68 | <0.001 |
| Piece 6 (vs. Piece 1) | -0.35 | 0.05 | 3280 | -6.52 | <0.001 |
| Piece 7 (vs. Piece 1) | -0.65 | 0.05 | 3280 | -11.90 | <0.001 |
| Piece 8 (vs. Piece 1) | -0.64 | 0.05 | 3280 | -11.81 | <0.001 |
| Standing (vs. Sitting) | 0.53 | 0.11 | 66 | 4.88 | <0.001 |
| Urge to Dance | 0.16 | 0.05 | 66 | 2.95 | 0.004 |
| Being Familiar With the Music | 0.04 | 0.05 | 66 | 0.79 | 0.432 |

**Table S14.** Overview of M13. HLM with movement entrainment as the dependent variable.

| M13 | | | | | |
|---|---|---|---|---|---|
| Value | Estimate | *SE* | *df* | *t* | *p* |
| (Intercept) | 0.27 | 0.09 | 3280 | 2.88 | 0.004 |
| Arm (vs. Torso) | 0.35 | 0.03 | 3280 | 12.73 | <0.001 |
| Half Time Level (vs. BPM Level) | -0.34 | 0.03 | 3280 | -10.19 | <0.001 |
| Double Time Level (vs. BPM Level) | -0.26 | 0.03 | 3280 | -7.76 | <0.001 |
| Piece 2 (vs. Piece 1) | -0.85 | 0.05 | 3280 | -15.62 | <0.001 |
| Piece 3 (vs. Piece 1) | -0.73 | 0.05 | 3280 | -13.46 | <0.001 |
| Piece 4 (vs. Piece 1) | -0.47 | 0.05 | 3280 | -8.66 | <0.001 |
| Piece 5 (vs. Piece 1) | -0.85 | 0.05 | 3280 | -15.68 | <0.001 |
| Piece 6 (vs. Piece 1) | -0.35 | 0.05 | 3280 | -6.52 | <0.001 |
| Piece 7 (vs. Piece 1) | -0.65 | 0.05 | 3280 | -11.90 | <0.001 |
| Piece 8 (vs. Piece 1) | -0.64 | 0.05 | 3280 | -11.81 | <0.001 |
| Standing (vs. Sitting) | 0.55 | 0.11 | 66 | 5.17 | <0.001 |
| Urge to Dance | 0.14 | 0.05 | 66 | 2.65 | 0.010 |
| Live Experience | 0.10 | 0.05 | 66 | 1.99 | 0.051 |

**Table S15: Preliminary Study: Set list**

| Performer | Piece title | Covered piece by | BPM | Comment |
|---|---|---|---|---|
| Solo Performances | Just Another Day | Lady Gaga | 82 | |
| | Girl With One Eye | Florence + the Machine | 82 | |
| | What a Wonderful World | Louis Armstrong | 82 | |
| | Your Song | Elton John | 62 | |
| | Strange | Celeste | 68 | |
| | New York | Paloma Faith | 86 | |
| Band 1 | Smells Like Teen Spirit | Nirvana | 122 | |
| | Emily | Adam Green | 128 | |
| | Wicked Game | Chris Isaak | 108 | |
| | Song 2 | Blur | 126 | |
| | *Encore:* Song 2 | Blur | 128 | |
| Band 2 | Highway to Hell | AC/DC | 124 | |
| | I shot the Sheriff | Bob Marley | 94 | |
| | Learn To Fly | Foo Fighters | 132 | |
| | *Encore:* Highway to Hell | AC/DC | 126 | |
| Band 3 | Boulevard of Broken Dreams | Green Day | 92 | |
| | Summer Wine | Lee Hazlewood & Nancy Sinatra | 136 | |
| | Thunderstorm | Self-written by guitarist Mascha | 148 | |
| | *Encore:* Boulevard of Broken Dreams | Green Day | 90 | |
| Band 4 | Talkin' Bout a Revolution | Tracy Chapman | 108 | |
| | Creep | Radiohead | 94 | |
| | Free Fallin' | Tom Petty | 94 | |
| | *Encore:* Free Fallin' | Tom Petty | 94 | |
| Brass Band | *Ein Prosit der Gemütlichkeit* | *Georg Kunoth* | *Strongly fluctuating tempi* | *Not used for analysis* |
| | *Happy Music* | *James Last* | *Strongly fluctuating tempi* | *Not used for analysis* |
| | Hit me with your best shot | Pat Benatar | 132 | |
| | Soon May the Wellerman Come | Sea Shanty | *Strongly fluctuating tempi* | *Not used for analysis* |

| | | | | |
|---|---|---|---|---|
| | Teenage Dirtbag | Wheatus | 104 | |
| | High Hopes | Panic! At the Disco | 144 | |
| | Bella Ciao / Havana (Medley) | trad. / Camila Cabello | *Strongly fluctuating tempi* | *Not used for analysis* |
| | Maxglaner Marsch | Robi Reiser | 152 | |
| | Feel It Still | Portugal. The Man | 144 | |
| | Drink doch eine met | Fred Hoock | 69 | |
| | Cordula Grün | Die Draufgänger | 134 | |
| | Interlude / Choral | | 60 | |
| | Mama Laudaaa | Almklausi & Specktakel | 134 | |
| | Angels | Robbie Williams | 70 | |
| | Teenage Dirtbag | Wheatus | 100 | |

**Table S16: Main Study: Set list**

| Piece title | Arranged by | BPM (concert 1) | BPM (concert 2) |
|---|---|---|---|
| In The Stone | Paul Murtha | 112 | 108 |
| Rainy Day in Vancouver | Chris Walden | 86 | 82 |
| Eleanor Rigby | Eric Richards | 140 | 136 |
| Too Close for Comfort* | Gordon Goodwin | 148 | 156 |
| Cancion de Cuna | Gabriel Peréz | 116 | 108 |
| Libertango | Paul Murtha | 140 | 138 |
| Mr & Mrs Adonis* (‘rehearsal’) | Mc Coy Mrubata, as recorded by “The Little Giants“ | 104 | 106 |
| Mr & Mrs Adonis* (with audience sing along) | Mc Coy Mrubata, as recorded by “The Little Giants“ | 104 | 108 |

** with vocals*

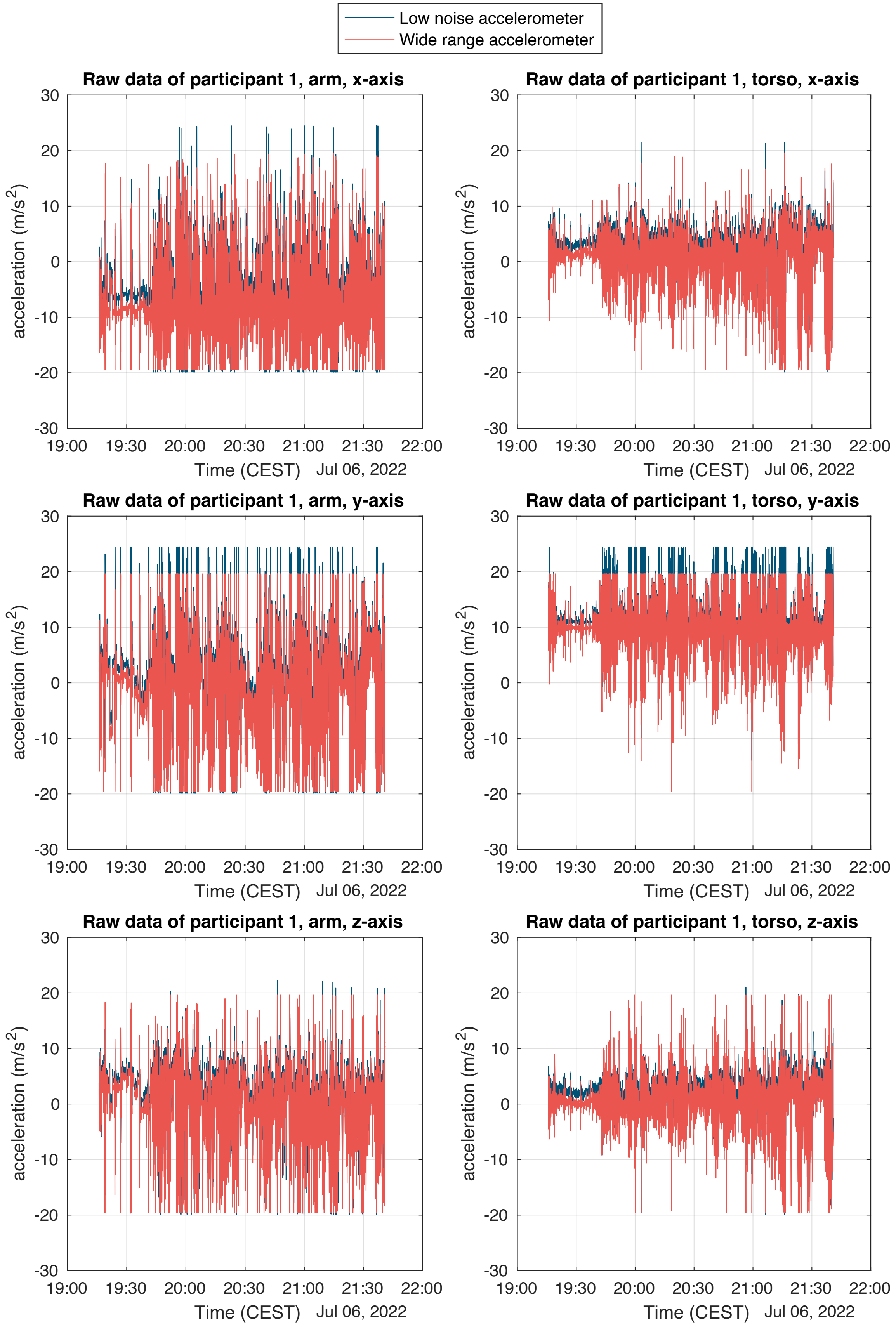


**Figure S17.** Raw data of the low noise accelerometer and the wide range accelerometer of participant 1 in the preliminary study for each location of measurement (arm and torso) and each axis (x-, y-, and z-axis).
Note: The lack of accelerometer calibration does not impact our analysis, as we are

interested in movement frequency and relative amplitude, but not absolute acceleration values.

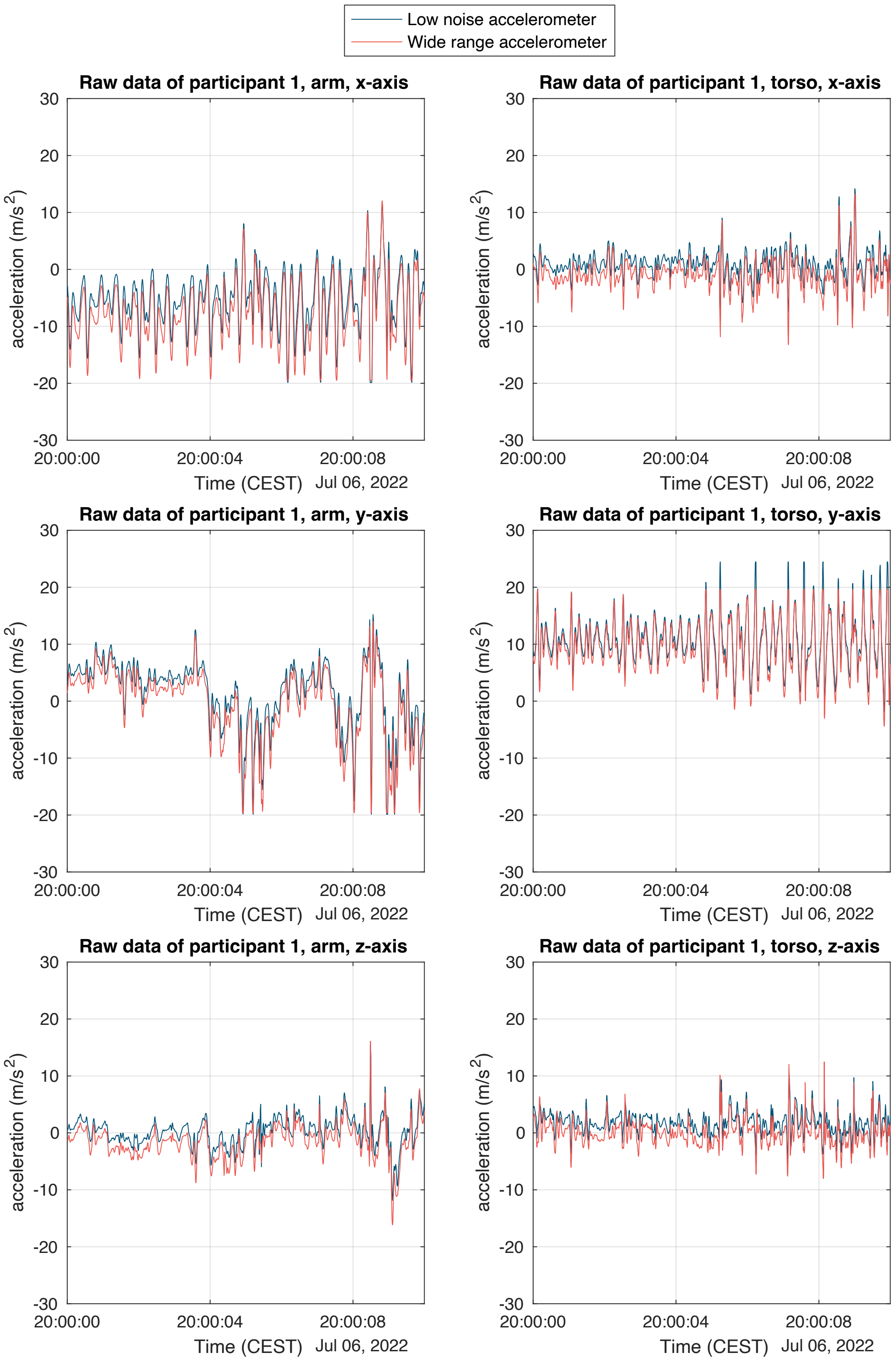


**Figure S18.** Raw data of the low noise accelerometer and the wide range accelerometer of participant 1 in the preliminary study for each location of measurement (arm and torso) and each axis (x-, y-, and z-axis) after interpolation. Detailed view of 10 seconds.

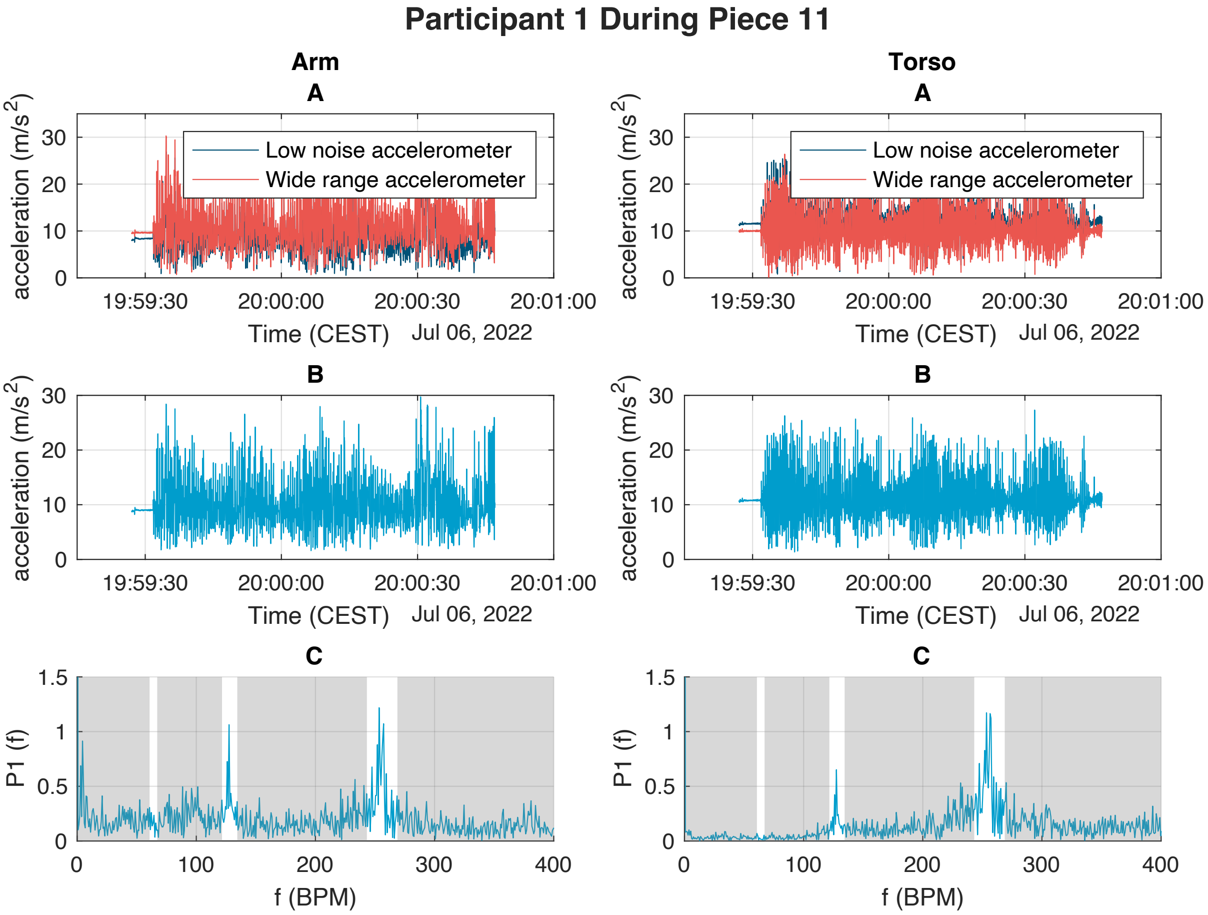


**Figure S19. A:** Combined x-, y-, and z-axis (after calculating the square root of the sum of the squares) of participant 1 in the preliminary study for each location of measurement (arm and torso) and both the low noise accelerometer and the wide range accelerometer during piece 11. **B:** Combined x-, y-, and z-axis (after calculating the square root of the sum of the squares) and combined data of the low noise and the wide range accelerometer of participant 1 in the preliminary study for each location of measurement (arm and torso) during piece 11. **C:** Single-Sided Amplitude Spectrum of the frequencies of the acceleration data of participant 1 in the preliminary study for each location of measurement (arm and torso) during piece 11. The BPM value of the piece was 128. The areas around the half-time level (64 BPM), standard beat level (128 BPM) and double-time level (256) with a tolerance range of ±5% are highlighted in white in the background.

**S20.** Results from the Questionnaires in the Main Study

Attending the concert alone or with another person: *Are you attending the concert today in company?* [orig.: *Besuchen Sie das Konzert heute in Begleitung?*]:
Concert 1: 82% in company, 18% alone
Concert 2: 73% in company, 27% alone

Liking the concert: *All in all, I found the concert...* [orig.: *Das Konzert alles in allem fand ich...*] (1 – 5 Likert scale, 1 = very bad, 5 = very good):
Concert 1: $M = 4.39$, $SD = 0.63$
Concert 2: $M = 4.24$, $SD = 0.83$

Liking jazz music: *Please indicate your basic preference for each of the following genres using the scale provided: Jazz* [orig.: *Bitte geben Sie Ihre Präferenzen für jedes der folgenden Musikgenres anhand der gegebenen Skala an: Jazz*]
(1 – 7 Likert scale, 1 = dislike strongly, 7 = like strongly):
Concert 1: $M = 5.04$, $SD = 1.75$
Concert 2: $M = 5.10$, $SD = 1.63$

Empathy[1] (index of four questions on 1 – 5 scales):
Concert 1: $M = 15.25$, $SD = 2.66$
Concert 2: $M = 15.63$, $SD = 3.17$

Dance Sophistication Index[2] (1 – 7 Likert scales, 1 = completely disagree, 7 = completely agree; *Items that were reverse-coded):
*Participatory Dance Experience*
P1: Body Awareness: *I am not very coordinated.* [orig.: *Ich bin nicht sehr geschickt, wenn ich mich bewege.*]*
Concert 1: $M = 4.89$, $SD = 1.77$
Concert 2: $M = 3.90$, $SD = 1.67$
P2: Social Dancing: *I like dancing in front of people.* [orig.: Ich mag es vor anderen Leuten zu tanzen.]
Concert 1: $M = 3.25$, $SD = 1.82$
Concert 2: $M = 3.67$, $SD = 2.16$
P3: Urge to Dance: *When I hear a great track, it just makes me want to dance.* [orig.: *Wenn ich ein tolles Musikstück höre, muss ich einfach anfangen zu tanzen.*]
Concert 1: $M = 4.43$, $SD = 2.01$
Concert 2: $M = 4.27$, $SD = 2.05$
P4: Dance Training: *I have had formal training in any dance style for: 0 years; 0.5 years; 1 year; 2 years; 3 years; 4 – 6 years; 7 or more years* [orig.: Ich hatte formales Training für einen bestimmten Tanzstil für … Jahre]
Concert 1: $M = 2.14$, $SD = 1.82$
Concert 2: $M = 2.39$, $SD = 1.94$
*Observational Dance Experience: If I had to choose, I'd rather watch a theater play than a dance performance.* [orig: Wenn ich die Wahl hätte, würde ich mir lieber ein Theaterstück als eine Tanzaufführung angucken.]*
Concert 1: $M = 4.18$, $SD = 1.59$
Concert 2: $M = 3.98$, $SD = 1.67$

PANAVA[3] (mean indices of 1 – 7 scales):
Positive Activation:
Concert 1: *M* = 4.92, *SD* = 0.90
Concert 2: *M* = 4.50, *SD* = 1.12
Negative Activation:
Concert 1: *M* = 2.53, *SD* = 0.90
Concert 2: *M* = 2.79, *SD* = 1.02
Valence:
Concert 1: *M* = 5.91, *SD* = 0.75
Concert 2: *M* = 5.27, *SD* = 1.36

Being familiar with the music: *How familiar were you with the music you heard tonight?* [orig.: *Wie vertraut waren Sie mit der heute Abend gehörten Musik?*] (1 – 4 Likert scale, 1 = very much, 4 = not at all):
Concert 1: *M* = 2.39, *SD* = 0.99
Concert 2: *M* = 2.44, *SD* = 1.00

Live experience: *I found experiencing the musicians live...* [orig.: *Die Musiker:innen live zu erleben fand ich...*] (1 – 5 Likert scale, 1 = very bad, 5 = very good):
Concert 1: *M* = 4.57, *SD* = 0.51
Concert 2: *M* = 4.46, *SD* = 0.67


1 Paulus, C. *Der Saarbrücker Persönlichkeitsfragebogen SPF (IRI) zur Messung von Empathie: Psychometrische Evaluation der deutschen Version des Interpersonal Reactivity Index*. https://doi.org/10.23668/psycharchives.9249 (2009).

2 Rose, D., Müllensiefen, D., Lovatt, P. & Orgs, G. The Goldsmiths Dance Sophistication Index (Gold-DSI): A psychometric tool to assess individual differences in dance experience. *Psychol. Aesthet. Creativity Arts* **16**, 733–745. https://doi.org/10.1037/aca0000340 (2022).

3 Schallberger, U. *Kurzskalen zur Erfassung der positiven Aktivierung, negativen Aktivierung und Valenz in Experience Sampling Studien (PANAVA-KS). Theoretische und methodische Grundlagen, Konstruktvalidität und psychometrische Eigenschaften bei der Beschreibung intra- und interindividueller Unterschiede. Forschungsberichte aus dem Projekt: „Qualität des. Erlebens in Arbeit und Freizeit“* **6**. https://www.psychologie.uzh.ch/dam/jcr:00000000-4a5f-c2e5-0000-000006e4f6b9/PANAVA_05.pdf (2005).
